# Prehospital EKG evaluation in Rio de Janeiro ambulances

**DOI:** 10.1186/cc12199

**Published:** 2013-03-19

**Authors:** RV Vasconcellos, FE Erthal, RV Vargas

**Affiliations:** 1 Instituto Nacional de Cardiologia, Rio de Janeiro, Brazil

## Introduction

Rio de Janeiro's Fire Squad is responsible for EMS in the city. During 2010 we implemented 10 ambulances with EKG transmission capability in our city. Our intention was to access the prevalence of acute myocardial infarction in the prehospital setting.

## Methods

We used the Aerotel HeartView EKG system to acquire patient examination and a blackberry phone to transmit and receive the PDF EKG trace. The PDF comes with the cardiologist's interpretation from a remote hospital, the HCOR São Paulo.

## Results

**Figure 1:**
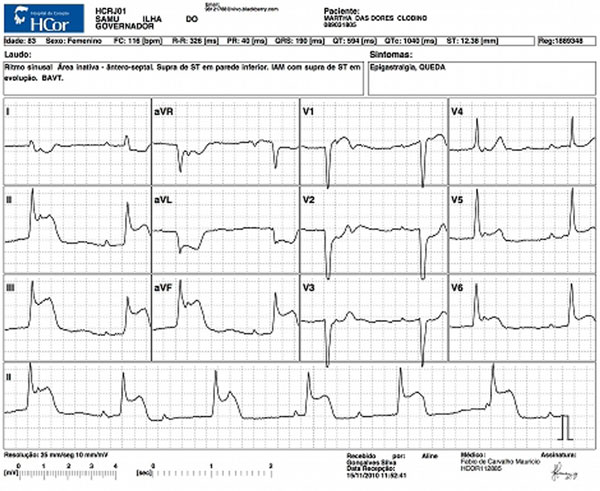
**Acute inferior MI with complete heart block**.

**Figure 2:**
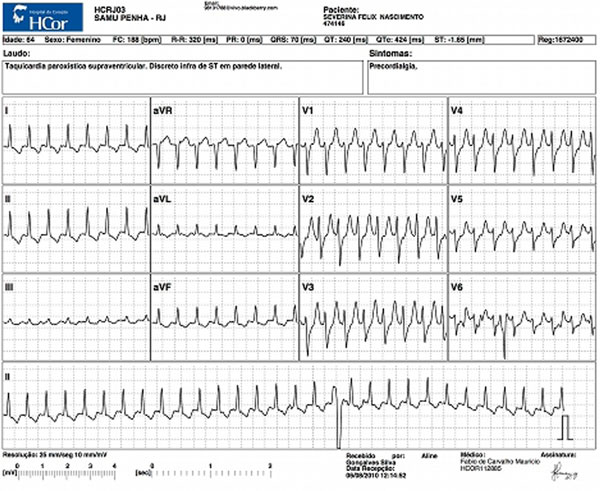
**Paroxysmal supraventricular tachycardia**.

We realized 503 EKG examinations in total. Of these, 248 (49%) had as the chief complaint chest pain, 101 (20%) shortness of breath, 47 (9%) syncope, 36 (7%) palpitation; other complaints were 15%. We detected 32 examinations (6.36%) with ST elevation MI and 44 examinations (8.75%) with ST depression. Atrial fibrillation was detected in 43 examinations (8.5%). See Figures 1 and 2.

## Conclusion

This experience gave us an idea of the prevalence for acute ST elevation MI in the prehospital setting, so that we can better develop our prehospital thrombolysis protocol and focus our training for cardiology care.
